# Maternal knowledge of childhood developmental milestones in Ashanti Region, Ghana

**DOI:** 10.11604/pamj.2024.49.117.40880

**Published:** 2024-12-11

**Authors:** Sheila Agyeiwaa Owusu, Ashura Bakari, Charles Kumi Hammond, Evans Otieku, Haruna Mahama, Cheryl Allen Moyer

**Affiliations:** 1 University for Development Studies, Department of Pediatrics and Child Health, Tamale, Ghana,; 2 Tamale Teaching Hospital, Department of Pediatrics and Child Health, Tamale, Ghana,; 3 Suntreso Government Hospital, Kumasi, Ghana,; 4 Kwame Nkrumah University of Science and Technology, Kumasi, Ghana,; 5 Komfo Anokye Teaching Hospital, Department of Child Health, Kumasi, Ghana,; 6 Institute of Statistical, Social, and Economic Research, University of Ghana, Legon, Ghana,; 7 Department of Public Health, Aarhus University, Aarhus, Denmark,; 8 University of Michigan Medical School, Ann Arbor, MI, USA

**Keywords:** Parental knowledge, childhood, developmental milestones, community-based

## Abstract

**Introduction:**

the ability of parents to recognize the age at which children attain developmental milestones helps in the early identification of delays and subsequent intervention to improve outcomes. However, there is a dearth of published evidence about parents´ knowledge of children´s developmental milestones in Ghana. The objective was to determine maternal knowledge of childhood developmental milestones (CDM) among a community-based sample of mothers of children under the age of five, identify the factors associated with CDM knowledge and the sources of information.

**Methods:**

a population-based cross-sectional study involving mothers of children less than five years resident in Akrofuom in the Ashanti Region of Ghana was selected in January 2023 using a multistage cluster sampling technique. Interviewer-based questionnaires were administered to eligible mothers. Knowledge of CDM and sources of CDM information were examined using descriptive statistics. Bivariate analysis was conducted to identify factors influencing CDM knowledge, and a multivariate logistic regression analysis was performed to evaluate the factors associated with overall CDM knowledge.

**Results:**

participants had low knowledge (19.3%) of all four domains of CDM. Approximately 40% of the participants reported receiving CDM information from relatives/friends and 14% from general health practitioners. Family income (p=0.01), participant level of education (p=0.04), and age of the first child (p=0.05) were significant influencing factors of CDM knowledge.

**Conclusion:**

the level of knowledge of mothers on CDM was low in all four domains emphasizing the need for healthcare workers and Pediatric Society Groups to increase their focus on educating parents, especially mothers, regarding knowledge of CDM.

## Introduction

The early years of a child´s life is considered critical in terms of brain development. During this period, any disruption by either an external stressor (such as illness or loss of a family member, lack of responsive caregiving, or low socioeconomic status of the family) or internal factors (such as molecular and cellular factors) increases the chance of developing a neurodevelopmental disorder or disability (NDD) [1-3]. Globally, approximately 10% of the population has some form of disability, as documented by the World Health Organization [4].

While the development of children influences their interactions and social bonding with family and friends, some studies show that parents´ knowledge about children´s development has a major influence on their interactions and growth, and is markedly associated with positive parenting efficacy [5]. A child´s ability to roll over, crawl, stand, walk, and run are crucial milestones in ensuring proper child development and neurological development, and parents´ awareness of possible signs of delay is vital for ensuring prompt interventions [6,7]. Published evidence suggests that parents with poor knowledge of child development overestimate the rate of child growth, potentially leading to inappropriate expectations, impatience, and intolerance [8-10]. Indeed, parents with a history of abusing or neglecting their children demonstrated low levels of knowledge of child development and were frustrated by the children's inability to comply with their expectations [11].

With the improvement in the diagnosis and treatment of many conditions during the early years of life, there has also been a significant increase in the number of children with chronic diseases. Children with chronic diseases are also prone to delayed neurodevelopment, psychosocial distress, and increased comorbidity with neurodevelopmental disorders [4,12]. Maternal knowledge about developmental milestones impacts their expectation of the age of attainment by the child, their care-seeking behavior, and their overall involvement in the general management of the child [13,14]. During the first 1000 days, there is rapid development of the brain; hence, any insults or deficiencies during this period have an adverse effect. However, based on the plasticity of the brain, early identification and interventions may help prevent adverse outcomes [15-17]. Improved access to reliable CDM information may help parents understand CDM, manage their expectations of the child, and seek appropriate help from health professionals [18,19].

Currently, there is a dearth of literature on parents´ knowledge of CDM in Ghana. Therefore, this study sought to generate scientific evidence with data to improve upon the lack of literature in the Ghanaian context. Thus, this study aimed to determine the maternal knowledge of childhood development milestones among a community-based sample of mothers of children under age five in the Akrofuom community in the Ashanti region, Ghana, assess sources of CDM information and identify factors associated with CDM knowledge to inform policymaking decisions.

## Methods

**Design:** a population-based cross-sectional study involving 150 parents, mostly mothers, was conducted using a multistage cluster sampling technique in January 2023.

**Study setting:** the study was conducted in Akrofuom. Akrofuom is the administrative capital of Akrofuom district. It is located in the southern part of the region. It lies within Latitude 40” North and 6 degrees 22” North and Longitude 1 degree West and 1 degree 38” West and has a total land area of 571 sq. km.

According to the 2021 population and housing census, the district's population is 49,291, with 26,315 males and 22,976 females. The district has a literacy rate of 64.8% of the population 6 years and older with a higher rate in males (69.7%) compared to females (59%). In the population of 15 years and above, agriculture is the main economic (66.8%) [20].

**Study participants:** the study population comprised mothers with under-five children resident in the Akrofuom community in the Ashanti Region of Ghana.

**Study variables:** the independent variables were maternal knowledge of developmental milestones, and the dependent variables were the parental age, educational level, marital status, and occupation.

**Inclusion and exclusion criteria:** only mothers with children less than 5 years old were enrolled in this study. Those who did not give consent or were not capable (due to health issues) of understanding the questionnaire were excluded.

**Sample size:** using Fisher´s formula for cross-sectional studies in populations above 10,000 (population in Akrofuom = 49,291), the sample size was calculated as:


$$n=\frac{Z^{2}pq}{d^{2}}=\frac{{\left(1.96\right)}^{2}\times 0.90\left(0.10\right)}{0.0025}=138$$

where n is the desired sample size, z is the standard normal deviation usually 1.96 corresponding to the 95% confidence level, p is the estimated proportion of the target population with a particular trait (80% in Riyadh by Aldayel *et al*.and 91.9% in Al-Ahsa by Noaim *et al*.) 90 % was considered in this study, q=1-p and d is the degree of accuracy desired set at 0.05 [13,21]. The estimated sample size of 138 was rounded to 150 to accommodate missing or incomplete data that may arise from data collection.

n = [(1,96)^2^x 0,90(0,10)/0,0025] = 138

**Sampling:** the sampling procedure for conducting the demographic and health surveys using the Ghana Statistical Service approach was followed. There are 11 enumeration areas (EA) in Akrofuom. A multistage cluster sampling technique was used to select the target samples. This was performed in three stages. Stage 1 involved the selection of five EAs using a simple random technique. Stage 2 involved a systematic listing of households to select approximately 30 households in each of the five EAs. For households to be selected, there must be at least a parent with a child under 5 years. Stage 3 involved selecting and interviewing eligible parent(s) from each identified household. Parents who did not consent to the study or were not in the right frame of mind to answer the questions were excluded.

**Data collection, processing, and analysis:** with permission, we used an adapted version of Aldayel *et al*. questionnaire to assess Parental Knowledge of children´s Developmental milestones [13]. The questionnaire included 17 items that assessed parents´ knowledge of children´s developmental milestones in four domains. This included 4 questions about physical development (walking, crawling, reaching for objects, dressing by themselves), 3 questions about cognitive development (engaging in pretend /fantasy play, following simple instructions, counting), 5 questions about social development ( parallel play, sharing toys, playing alone for an hour, having best friends, showing empathy), and 5 questions about emotional development (exerting independence, recognizing others´ emotions, differential cries, bonding with a caregiver, advocating for fairness). Seven additional questions assessed mothers´ sources of knowledge and their frequency of source use. Sociodemographic variables such as age, sex, parents´ level of education, occupation of parents, and age of the first child were also assessed. The survey was piloted among 10 randomly selected participants at Suntreso Government Hospital in Kumasi during their child welfare clinic to ensure that the survey was understandable and easy for mothers to complete.

Two research assistants (RAs) were trained by the principal investigator to approach potential respondents, obtain informed consent, administer the questionnaire, and accurately enter the data using a Google document on a hand-held, electronic tablet. Data collected in the field were imported into Microsoft Excel (Redmond, WA, USA) for cleaning.

Data was analyzed using STATA version 16. Descriptive statistics were used to evaluate participants´ knowledge of childhood development milestones and their sources of information. We performed a stepwise analysis. To be knowledgeable in the physical developmental milestones of a child, a participant must obtain a correct score of at least two out of the four subdomains. The same applied to the measurement of emotional developmental milestones. For cognitive developmental milestones, a correct score of at least one out of the three subdomains (33%) was deemed knowledgeable. For social developmental milestones, a correct score of at least two out of the five (40%) subdomains was deemed knowledgeable.

The overall CDM knowledge was estimated as the cumulative CDM knowledge for each domain and transformed into an overall binary outcome (1=knowledgeable; 0=not knowledgeable). Because each domain has sub-measurement indicators altogether making 16 indicators, we defined knowledgeable to mean a score of 40% of the 16 indicators. Thus, a correct score of less than 7 out of the 16 total indicators means the participant is not knowledgeable or has low CDM knowledge. Multivariate logistic regression was used to evaluate the odds ratio (OR) of factors associated with participants´ overall knowledge of CDM. The regression analysis was preceded by chi-squared analysis to evaluate the association between categorical variables and examine associated patterns. A p-value of <0.05 was taken for statistical significance.

**Ethical approval and consent to participate:** this study received permission from the Ashanti Regional Health Directorate, community entry approval by the District Health Directorate and community leaders as well as ethics approval by the Ethics Review Committee of the Kwame Nkrumah University of Science and Technology (KNUST) (CHRPE/AP/734/22). The details of the study were explained to participants after which informed consent was obtained before their participation.

## Results

**Description of participants socio-demographics:** a total of 145 mothers with children under age five were studied. Though the sampling included five fathers (male participants), we limited the analysis to only mothers (female participants) as they constituted 96.7% of the sample. Regarding ethnicity, most of them were Akan (61.4%, n=89). Approximately two-thirds of the study sample were less than 35 years old (69.7%, n=101), and the majority had their first child when they were 15-19 years old (40.7%, n=59). Most of the participants were in gainful employment (53.8%, n=78) and had completed senior high school (74.5%, n=108), while less than eight percent (6.2%, n=7) had a monthly household income of more than 3000 Ghana cedis, equivalent to USD1414 in purchasing power parity in International United States Dollars. About one-third of the participants were single parents (36.6, n=53), and most had children less than one-year-old (65.3%, n=98) (Table 1).

**Table 1:** participants’ socio-demographic characteristics

Category	Variable	N (%)
**Ethnicity of participant**	Akan Ewe Fanti Kusasi Others*	89 (61.4) 11 (7.6) 11 (7.6) 9 (6.2) 25 (17.2)
**Age (in complete years)**	15 - 24 25 - 34 35 - 44 >45	31 (21.4) 70 (48.3) 30 (20.7) 14 (9.7)
**Age when your first child was delivered**	15yrs - 19yrs 20yrs - 24yrs 25yrs - 29yrs 30yrs - 33yrs	59 (40.7) 55 (37.9) 27 (18.6) 4 (2.8)
**Working status**	Self-employed Employee Student Unemployed	78 (53.8) 2 (1.4) 45 (31.0) 20 (13.8)
Monthly household income (GHS)**	≤500 501 - 999 1000 - 1499 1500 - 2000 2001 - 3000 >3000 Don’t’ know	29 (25.7) 16 (14.2) 19 (16.8) 9 (8.0) 6 (5.3) 7 (6.19) 27 (23.9)
**Highest level of education obtained**	None Basic school High school Tertiary (Diploma, Degree) Higher tertiary (Master/Doctorate)	23 (15.9) 7 (4.8) 108 (74.5) 5 (3.4) 2 (1.4)
**Marital status**	Single Married Divorce Widow	53 (36.6) 87 (60.0) 3 (2.0) 2 (1.4)
Number of children you have under-5 years **	1 2 3	94 (65.3) 44 (30.6) 6 (4.1)
**Age of first child**	Less than 1 year 1yr - 4yrs 5yrs - 10yrs 11yrs - 15yrs 16yrs and above	11 (7.6) 37 (25.5) 43 (29.7) 25 (17.2) 29 (20.0)
**Gender of first child**	Female Male	74 (51.0) 71 (49.0)
**Residence of child with parent**	Full-time with parent Part-time with parent Not with parent at all	96 (65.3) 30 (20.7) 19 (13.1)
**Have a child with special need**	Yes No	1 (.07) 144 (99.3)
**Household size**	95% Confidence interval	5 (95%CI. 4.6 – 5.5)

* Comprising 9 other ethnic groups, ** Some participants did not answer the question

**Stated knowledge of CDM by participants:** we assessed mothers´ perceived knowledge of childhood developmental milestones across four domains and found that only an estimated 19.3% of the participants were knowledgeable based on our measurement indicators. For instance, concerning a child´s physical development, only 30.4% of the respondents were knowledgeable in at least two out of the four sub-measurement indicators. Regarding the emotional development of a child, only an estimated 16.6% of the participants were knowledgeable compared to 18.6% for cognitive development and 14.5% for social development. Table 2 shows how participants responded to CDM knowledge questions for each domain and the estimated proportion of participants who were knowledgeable in CDM based on our scoring criteria.

Approximately the same proportion of the participants knew that a child was supposed to begin counting from 24 to 36 months of age. Participants´ knowledge regarding the cognitive and social development milestones of a child was low compared to the remaining two domains. Overall, only 19.3% (n=28) of the participants had adequate CDM knowledge cutting across all four domains (Table 2).

**Table 2:** perceived parental knowledge of childhood development milestone

Developmental domain	Correct answer	Correct response N (%)	Incorrect response N (%)
**Physical development**			
Reach for objects*	4-6 months	41 (28.3)	104 (71.7)
Crawl**	7-12 months	48 (33.8)	96 (66.2)
**Walk**	12-18 months	51 (34.5)	95 (65.5)
Dress themselves*	24-36 months	37 (24.8)	109 (75.2)
**Sub overall knowledge**		44 (30.4)	101 (69.6)
**Emotional development**			
**Differential cries**	4-6 months	30 (20.0)	116 (80.0)
Recognise other's emotions*	7-12 months	13 (9.0)	132 (91.0)
**Exert independence**	12-18 months	36 (24.1)	110 (75.9)
Advocate for fairness*	60-72 months	16 (11.1)	129 (88.9)
**Sub overall knowledge**		24 (16.6)	121 (83.4)
**Cognitive development**			
**Engage in pretend/fantasy play**	12-18 months	20 (13.1)	126 (86.9)
Follow simple instructions*	12-18 months	11 (6.9)	135 (93.1)
Begin counting*	24-36 months	51 (35.2)	94 (64.8)
**Sub overall knowledge**		27 (18.6)	118 (81.4)
**Social development**			
Parallel play*	18-24 months	26 (17.3)	119 (82.1)
**Share toys**	36-60 months	28 (18.5)	117 (80.7)
**Play alone for 1 hour**	36-60 months	17 (11.3)	128 (88.3)
Show empathy*	60-72 months	11 (7.3)	134 (92.4)
Have best friends*	60-72 months	23 (15.3)	122 (84.1)
**Sub overall knowledge**		21 (14.5)	124 (85.5)
**Estimated overall CDM knowledge**		28 (19.3)	117 (80.7)

* 1 participant had no idea and did not answer the question **2 participants had no idea and did not answer the question

**Factors associated with CDM knowledge among participants:** three sociodemographic factors were significantly associated with participants´ overall knowledge of CDM. They included household income (p=0.01), received formal education formal education (p=0.04), and age of the participants´ first child (0.05) (Table 3).

**Table 3:** association between respondent’s sociodemographic characteristics and knowledge of childhood developmental milestones

Category	Variable	X2 (p-value)
**Ethnicity of participant**	Akan Ewe Fanti Kusasi Others	1.6 (0.78)
**Age (in complete years)**	15yrs - 24 25 - 34 35 - 44 >45	5.4 (0.15)
**Age when your first child was delivered**	15yrs - 19yrs 20yrs - 24yrs 25yrs - 29yrs 30yrs - 33yrs	1.5 (0.67)
**Working status**	Self - employed Employee Student Unemployed	1.8 (0.56)
**Monthly household income (GHS)**	≤500 501 - 999 1000 - 1499 1500 - 2000 2001 - 3000 >3000 Don’t’ know	16.4 **(0.01)**
Received formal education*	Yes (n=126) No (n=25)	9.6 **(0.04)**
**Marital status**	Single Married Divorce Widow	2.4 (0.49)
**Number of children you have under-5 years**	1 2 3	1.8 (0.42)
**Age of first child**	Less than 1 year 1yr - 4yrs 5yrs - 10yrs 11yrs - 15yrs 16yrs and above	31.8 **(0.05)**
**Gender of first child**	Female Male	4.8 (0.45)
**Residence of child with parent**	Full-time with parent Part-time with parent Not with parent at all	3.2 (0.21)
**Household size**	≤4 persons >4 persons	3.5 (0.60)

* Re-coded as a binary outcome for a better precision estimate

Adjusted results in Table 4 using logistic regression estimates indicate that households with monthly income between GHS1500 and GHS2000 (PPP adjusted: USD 707 - USD 943) were 6.5 times more likely to have CDM knowledge than the reference group that earned less GHS500 (OR: 6.5, 95%CI: 2.0 - 35.7). Furthermore, participants who had received any form of formal education were approximately 1.8 times more likely to have CDM knowledge than those without formal education (OR: 1.8, 95%CI: 1.3 - 4.8). Likewise, participants were 1.5 times more likely to have CDM knowledge if their first child was between 5 and 10 years old than if the child was less than a year old (OR: 1.5, 95%CI: 1.2 - 5.5).

**Table 4:** adjusted odds ratio and 95% confidence intervals (95%CI) of predictors for childhood developmental milestones

Sociodemographic factors	OR	P-value
**Household income (GHS)** <500 (ref) 501 - 999 1000 - 1499 1500 - 2000 2001 - 3000 >3000	- 1.6 (0.4 - 7.2) 0.5 (0.2 - 2.1) 6.5 (2.0 - 35.7) 1.4 (0.2 - 10.2) 0.8 (0.1 - 5.2)	0.49 0.34 0.03 0.75 0.83
**Received formal education** No (ref) Yes	- 1.9 (1.3 - 4.8)	0.01
**Age of first child** Less than 1 year (ref) 1yr - 4yrs. 5yrs - 10yrs 11yrs - 15yrs 16yrs and above	- 0.5 (0.2 - 1.8) 1.5 (1.2 - 5.5) 0.3 (0.1 - 2.2) 0.3 (0.1 - 1.7)	0.47 0.02 0.68 0.66

**Stated sources of CDM information by participants:** concerning participants´ sources of CDM information, they were presented with seven sources of information to indicate where they received information regarding the CDM. In descending order of magnitude, the analysis shows that approximately 41% of the participants received CDM information from relatives and friends always, 14.5% received the same information from general practitioners always, Parenting books/magazines (4.8%), Internet sources (2.1), social media broadcasts (1.4%), and parenting seminars/course and social media were the least identified sources of CDM information among participants (Table 5).

**Table 5:** sources of information used by parents concerning childhood developmental milestones

Information source	Response	N (%)
**Relatives and friends (n=145)**		
	Always	59 (40.7)
	Usually	20 (13.8
	Rarely	10 (6.9)
	Never	56 (38.6)
**General practitioner (n=145)**		
	Always	21 (14.5)
	Usually	44 (30.3)
	Rarely	21 (14.5)
	Never	59 (40.7)
**Parenting books and magazines (n=145)**		
	Always	7 (4.8)
	Usually	33 (22.8)
	Rarely	16 (11.0)
	Never	89 (61.4)
**Internet websites (n=143)**		
	Always	2 (1.4)
	Usually	8 (5.6)
	Rarely	7 (4.9)
	Never	126 (88.1)
**Social media broadcast (n=144)**		
	Always	1 (0.7)
	Usually	11 (7.6)
	Rarely	12 (8.3)
	Never	120 (83.3)
**Parenting seminars and courses (n=145)**		
	Always	1 (0.7)
	Usually	17 (11.7)
	Rarely	18 (12.4)
	Never	109 (75.2)
**Television shows**		
	Always	3 (2.1)
	Usually	39 (26.9)
	Rarely	26 (17.9)
	Never	77 (53.1)

## Discussion

Parental knowledge of age-specific developmental milestones is essential for raising healthy children. Knowing appropriate developmental milestones can prompt parents to seek care early when there is a delay and can avoid unnecessary pressure on children to meet milestones before they are ready. This study assessed parental knowledge of childhood developmental milestones among a community-based sample of parents of children under the age of five, the factors associated with greater levels of knowledge, and the sources of information.

The findings show that there is a low level of knowledge (19.3%) regarding all four domains of childhood developmental milestones (CDM) examined. This is similar to the findings of Aldayel *et al*. (20%) in a study done in Riyadh, Saudi Arabia. However, this contrasts with other studies in which parental knowledge of CDM was high, except for the knowledge regarding a child´s cognitive and social development [9,11,13]. A possible explanation could be that this study was community-based and was conducted in a rural setting where a small proportion of inhabitants received tertiary education and had little or no knowledge of CDM, except for their parenting experiences. In contrast, Safadi *et al*. [9] recruited study participants from the Ministry of Health and Maternity and Child Health Care Centers (MCH), whereas Aldayel *et al*. [13], recruited participants from Saudi Telecom Company´s database in Riyadh, which is an urban setting and perhaps with more educated parents (participants) who have improved access to CDM information. In addition, more than a third (40.4%) of our respondents had their first child when they were younger than 20 years. Delayed parenthood has been documented to be associated with a high level of knowledge since most of these mothers are likely to be more educated. In the younger age group, most mothers have limited knowledge about parenting, and therefore continued support from family and healthcare providers is needed to augment this knowledge gap [13]. It is, therefore, necessary to streamline health education messages not only to expectant mothers but also to target senior high schools and particularly rural communities with messages that they can easily appreciate and apply.

With the assessment of sociodemographic characteristics, our study found a significant association between parental education and household income, which indirectly affects the cognitive development of the child, as reported in two other studies [14,17]. Unlike other studies [9,11,13], we found that households with monthly income between GHS1500 and GHS 2000 were 6.5 times more likely to have CDM knowledge than the reference group that earned less than GHS500. Participants who had received any form of formal education were also approximately 1.8 times more likely to have CDM knowledge than those without formal education. Therefore, education and financial empowerment must be considered in the overall measures to improve the knowledge of parents, as reported in another study in Canada [19]. An interesting finding was that participants were 1.5 times more likely to have CDM knowledge if their first child was between 5 and 10 years old than if the child was less than a year old. This is likely because most of these participants were multiparous and had more experience with children, which is congruent with the findings of Alkhazrajy *et al*. [15], who showed that there was an association between the number of children and parental knowledge.

Compared with other related studies, there were similarities and differences in where participants sought CDM information. For example, less than one-fifth of participants said they received CDM information from physicians, which contrasts with the findings of Rickhy *et al*.who found that doctors, books, and nurses were the most common sources of CDM information [11]. Although there is widespread information on children´s development in both electronic and print media, people still do not access this information either because they are not aware, unable to access it, or do not appreciate the content [11]. This allows healthcare workers to strengthen their health education measures, especially in rural communities where some parents may have low levels of formal education and will not be able to obtain information from print media. It is necessary to elaborate on CDM during each visit throughout pregnancy and visits to child welfare clinics.

This study had several notable strengths, including a robust sampling design and the use of a previously validated instrument to assess knowledge of childhood developmental milestones.

**Limitations:** this study has two limitations. First, the study focused on mothers with children under five years, because they were most easy to contact. Akrofuom is also a predominantly farming community and the timing of the interviews did not permit fathers to be included in the study. Future studies can consider a different time for the interviews to get a study population involving both parents. This study therefore cannot conclude that fathers (men) also have low levels of CDM knowledge because male respondents were not representative of fathers with fewer than five children in the study population. In addition, there is the possibility of social desirability bias, where parents might report what they believe researchers want to hear. However, given that the responses were based on age ranges, we do not believe that social desirability bias was a significant problem in this study.

## Conclusion

Knowledge of childhood developmental milestones in the Akrofuom community in the Ashanti region of Ghana is generally low, which can prevent parents from seeking care if there is developmental delay. It is also possible that parents have unrealistic expectations for their children. To this end, it is important to empower parents to identify both appropriate and inappropriate childhood development characteristics. The family´s income and level of education play important roles in the knowledge of these milestones, and it is therefore important to strengthen programs that seek to enhance enrollment in schools, such as the school feeding program and free senior high school initiatives. The findings from this study suggest that more emphasis should be placed on the age of attainment of these milestones. It is therefore important that the content of educational sessions, especially during antenatal and postnatal visits and child welfare clinics be modified to project CDM and early childhood development/. Health advocacy groups and health professional groups such as the Pediatric Society of Ghana will need to heighten their educational sessions and target rural communities, as they may not be able to access the Internet or print media.

### What is known about this topic

- The population of children with chronic diseases is growing;
- Parental knowledge of illness affects care-seeking behavior and prevents anxiety.

### What this study adds

- Although much education is ongoing concerning child development, most parents are still not abreast with this information, especially in rural areas;
- Social determinant factors, such as education and family income, have an impact on parental knowledge of childhood developmental milestones;
- Healthcare workers and Pediatric Society Groups will need to intensify their education in rural communities.
